# Cycle training for children: Which schools offer it and who takes part?

**DOI:** 10.1016/j.jth.2015.07.002

**Published:** 2015-12

**Authors:** Anna Goodman, Esther M.F. van Sluijs, David Ogilvie

**Affiliations:** a Faculty of Epidemiology and Population Health, London School of Hygiene and Tropical Medicine, London WC1E 7HT, United Kingdom; b MRC Epidemiology Unit and UKCRC Centre for Diet and Activity Research (CEDAR), University of Cambridge School of Clinical Medicine, Box 285, Cambridge Biomedical Campus, Cambridge CB2 0QQ, United Kingdom

**Keywords:** Cycling, Cycle training, Children, Schools, Equity, Bikeability

## Abstract

**Purpose:**

The ‘Bikeability’ cycle training scheme, a flagship policy of the government in England, aims to give children the skills and confidence to cycle more safely and more often. Little, however, is known about the scheme׳s reach. This paper examined which schools offer Bikeability, and which children participate in cycle training.

**Methods:**

We used operational delivery data to examine which primary schools in England offered Bikeability. Predictors included the deprivation level of the student body and the local prevalence of cycling. We then examined cycle training participation using data from 6986 participants (age 10–11) in the nationally-representative Millennium Cohort Study. Parents reported whether their child had completed formal cycle training, along with other child and family factors. We used operational data to identify children whose school had previously delivered Bikeability.

**Results:**

55% of schools offered Bikeability to the cohort of children leaving primary school in 2012; this fell to 48% in schools in the top tenth for student deprivation. Among Millennium Cohort participants, 47% of children had completed cycle training; this proportion rose to 68% among children whose schools had offered Bikeability. In adjusted robust Poisson regression models, participation rates were lower among minority ethnic children, particularly South Asians; among children who played sport less often; and among children whose parents were poorer or less educated. The magnitude of these differences was largest among children whose schools had not offered Bikeability (all *p*≤0.02 for interaction, except for income where *p*=0.09), although trends in the same direction were observed in schools that had offered Bikeability.

**Conclusions:**

Offering high-quality cycle training free of charge in English schools reduced but did not eliminate inequalities in cycle training participation. Further promoting the scheme to parents and schools, particularly in deprived areas, would be expected to increase uptake and help reduce current inequalities in participation.

## Highlights

• Around half of children in England receive “Bikeability” cycle training in school.
• Schools with a more deprived student body offer Bikeability slightly less often.
• Cycle training participation is higher in children who are White, affluent or sporty.
• These differences in participation rates are smaller in schools offering Bikeability.
• Further promoting Bikeability could reduce but not necessarily eliminate inequalities.

## 1 Introduction

Promoting cycling, including promoting cycling among children, has in recent years moved up multiple policy agendas in a number of high income countries (The PEP, 2009, Australian Department of Health and Aging, 2010, Department for Transport, 2011, Department of Health and Department for Transport, 2010, Welsh Government, 2012, American Public Health Association, 2012). This reflects various factors, including the health benefits of increasing physical activity among children (Chief Medical Officers of England, Scotland, Wales, and Northern Ireland, 2011); the economic and social benefits of reducing the congestion and community severance associated with car-dominated transport systems (Woodcock and Aldred, 2008, Cabinet Office, 2009); and the environmental benefits of reducing the greenhouse gas emissions associated with motorised travel (Woodcock et al., 2009). The potential magnitude of these benefits is considerable, given the substantial proportion of motorised trips that could in theory be made by bicycle (Woodcock et al., 2013): for example, around two-thirds of short trips (≤2 km) by children are made by car and only 3% by bicycle (Department for Transport, 2013).

Fears about dangerous traffic and about children׳s ability to cycle safely on the road are reported by many children and parents as key reasons why children do not cycle more (Lorenc et al., 2008). One correspondingly popular (Granville et al., 2002, Ipsos MORI, 2010) means of promoting cycling is ‘cycling proficiency’ training for children. Such training is very common in several high-cycling European countries such as the Netherlands, Denmark and Germany, and is also fairly widespread (although more variable in its regional coverage) in some lower-cycling countries such as France, the United States, Australia and New Zealand (Pucher and Buehler, 2008, Pucher et al., 2010). In the UK, the Department for Transport substantially increased its support for such child cycle training through its launch of the Bikeability scheme in England in 2007. Aspiring to provide “cycling proficiency for the 21st century” (Department for Transport, 2012a, p. 6), this flagship scheme involves using schools to deliver high-quality on- and off-road cycle training during the final years of primary school. Typically children in primary school learn both how to ride a bicycle (level 1) and also how to make short journeys safely and confidently on local roads (level 2). Schools can offer the scheme free of charge, with costs covered by central and local government funding. Bikeability sessions are delivered by external organisations in collaboration with schools, and parents are asked for their permission for their child to take part.

The aim of Bikeability is to give children “the skills and confidence they need to cycle on today׳s roads…[and thereby to] encourage more children to cycle more often, more safely” (Frearson and Hewson, 2014, p. 7). Evidence as to the scheme׳s impact is currently strongest with respect to the first, more proximate, set of outcomes, with a recent controlled evaluation finding positive effects on skills such as hazard perception and on confidence (Hodgson and Worth, 2015). There is currently less evidence that Bikeability increases cycling frequency, although one ecological study (Department for Transport, 2012) and one individual-level pilot study report encouraging results (Frearson, 2013). In this respect the evidence base for the effectiveness of Bikeability mirrors the wider international literature, which provides reasonable evidence that child cycle training can have positive impacts on knowledge, skills or safety behaviour (reviewed in RoSPA (2001); Richmond et al. (2014), see also Ducheyne et al. (2014); Hooshmand et al. (2014)) but contains few studies of impacts on cycling frequency. The studies of these impacts that do exist are inconclusive, reporting mixed findings (Savill et al., 1996) or null results in small samples (Ducheyne et al., 2014).

The Bikeability evidence base also mirrors the wider literature in containing very little information as to which children actually receive cycle training; indeed, to our knowledge no study has been published that examines this. Coverage of the Bikeability scheme in England grew rapidly after 2007, but in the past few years uptake has flattened at around half of all children of the relevant age (*N*=250,000 children trained per year) (Department for Transport, 2014). Examining which schools and which children are receiving this training could help policymakers and practitioners to understand the current distributional impacts of Bikeability and could inform attempts to increase the reach of the scheme still further. It would also contribute to the wider international evidence base surrounding initiatives to promote cycling, which typically contains very little examination of the likely or actual social distribution of impacts (Yang et al., 2010, Ogilvie et al., 2004). Several commentators have highlighted this shortcoming as a source of concern (NICE, 2008, NICE, 2012, Marmot, 2010), particularly in light of calls for public policy to be delivered in ways that are equitable as well as evidence-based (Ståhl et al., 2006, Kahlmeier et al., 2010). This paper therefore aimed to combine recent school-level and child-level information from England in order to examine (1) which schools offer Bikeability training and (2) which children participate in cycle training.

## 2 Methods

### 2.1 School-level analyses: which schools offer Bikeability?

#### 2.1.1 Sample of schools

The Department for Education׳s ‘Edubase’ is a register containing information on all state-funded and private schools in England and Wales. Using Edubase, we identified all English primary schools that were open on 1st September 2011 and that contained both Year 5 and Year 6 pupils. This generated a list of 14,401 English primary schools, from which we excluded 1590 in London (as we did not have Bikeability delivery data for this region), giving a sample of 12,881 schools.

#### 2.1.2 Outcome: Bikeability offered by schools

We sought to identify all schools that had offered Bikeability cycle training to the cohort of children leaving primary school in 2012. For this we used operational data provided by the Department for Transport. Schools are encouraged to deliver Bikeability in the final year of primary school (Year 6, age 10–11), but a minority instead deliver the training a year earlier (Year 5, age 9–10). We therefore sought to identify all schools that had offered cycle training to the 2012 cohort of interest at some point during their final two years of school. We did this by identifying schools that either offered Bikeability to Year 5 children in the academic year 2010/11 or offered Bikeability to Year 6 children in the academic year 2011/12. In the case of some Year 5 delivery this involved approximating academic years using financial years, but sensitivity analyses indicated that the likely effect of this on our results was minimal (see Supplementary material for further details).

#### 2.1.3 Exposures: school-level characteristics

Edubase additionally provided data on the number of pupils in the relevant year group, and also on the proportion of children in the school receiving free school meals, a commonly-used marker of the deprivation of the student body. Both of these items of data were recorded in the school census collected in January 2012. Edubase also linked each school to the 2004 Rural and Urban Area Classification (Bibby and Shepherd, 2004), thereby defining its urban/rural status. Urban/rural status was defined as a three-level categorical variable: large urban areas with a population >10,000; smaller towns and fringe areas; and villages, hamlets and isolated dwellings

Using the schools׳ postcodes, we matched each school to its local authority and region of England. To establish area-level cycling prevalence, we also matched each school to its Middle Super Output Area; these are administrative areas with populations of around 5000, which is approximately the same scale as the catchment area of a primary school. We then assigned to each school the proportion of adults in the Middle Super Output Area who reported that cycling was their ‘usual, main mode’ of travelling to work in the United Kingdom (UK) census collected in March 2011. We have previously shown that this measure of commuter cycling provides a reasonable proxy for the total cycle mode share in an area, as calculated with reference to all trips made by adults aged over 16 (Goodman, 2013). Note that correspondingly fine-grained proxies for total cycling participation among children are not available, but the National Travel Survey provides evidence that adult modal share and child modal share are fairly highly correlated at a regional level (Pearson correlation coefficient 0.72) (Department for Transport, 2013).

#### 2.1.4 Statistical analysis

We used Poisson regression with robust standard errors clustered by local authority in order to examine the correlates of a school offering Bikeability. We used schools as our units of analysis and entered independent variables categorically as shown in Table 1. We used robust Poisson regression in preference to logistic regression because it provides an estimate of the risk ratio for common outcomes (Zou, 2004). By contrast, for common outcomes logistic regression generates odds ratios that are notably further from the null than the risk ratio, which may make results prone to misinterpretation. Our substantive findings were very similar when we instead fit multi-level logistic regression models of schools nested within local authorities nested within regions, to reflect the fact that local authorities contain multiple schools (an average of 44 schools per local authority) and regions contain multiple local authorities (an average of 37 local authorities per region). All statistical analyses were conducted using Stata 13.1, and we used ArcMap 10.0 to create the map shown in Supplementary material.

### 2.2 Child-level analyses: which children participate in cycle training?

#### 2.2.1 Sample of children

The Millennium Cohort Study (MCS) is a population-based birth cohort that has followed a sample of British children across five sweeps (Plewis, 2007, Hansen, 2014). It can be analysed to provide nationally-representative results either at the level of the UK, or at the level of individual countries such as England. The first sweep took place in 2001/02 when the children were around 9 months old; subsequent sweeps have happened in 2003/04, 2006, 2008 and 2012. The fifth sweep (age 10/11) successfully collected data on 13,403 children (51% of those eligible to participate in the first MCS sweep). We excluded children attending school outside England (*N*=4779) or in London (*N*=1322), where school-level information on Bikeability delivery was not available. We also excluded children not attending school (*N*=14) or in a non-standard school year (*N*=302), leaving a study population of 6986 children (3515 males; 50.3%) interviewed between January 2012 and August 2012.

Parents participating in MCS provided informed, written consent, and children provided oral assent for direct measurements (e.g. of height and weight). Ethical approval for the fifth sweep of the MCS was granted by the Yorkshire and Humber research ethics committee (Ref:11/YH/0203 (Hansen, 2014)). Ethical approval for our analyses was granted by the London School of Hygiene and Tropical Medicine׳s ethics committee (Ref: 7034).

#### 2.2.2 Outcome: child participation in cycle training

Parents completing MCS survey interviews were asked “Has [Child] ever done any formal cycling proficiency training such as ‘Bikeability’? Formal cycling proficiency training is delivered by a recognised trainer and includes tuition on the road” (response categories yes/no). This question was purposively developed in collaboration with the Department for Transport; no information is available regarding its reliability or validity.

#### 2.2.3 Exposures: child, family, area and school characteristics

Table 2 presents the potential predictors of whether the child had completed cycle training. The child and family characteristics were almost all provided in the fifth sweep of MCS, except for whether the child cycled to or from school at age 7, which was provided in the fourth sweep. All characteristics relied on parental report from one of the child׳s primary caregivers (58% mothers, 37% fathers, 5% other caregivers), and were obtained during computer-assisted face-to-face interviews (questionnaires available at www.cls.ioe.ac.uk/MCS). The only exception was weight status, which was derived using measures of height and weight taken by trained interviewers during the MCS interview, and defined using standard cut-points (Cole et al., 2000).

The urban/rural status of the child׳s home postcode was calculated in the same way as described above for the school-level analyses. The local prevalence of cycling to work in the 2011 UK census was calculated at the level of slightly smaller areas, namely the Lower Super Output Area of the child׳s home address (Lower Super Output Areas are administrative areas containing around 1500 individuals, and are commonly used to approximate the immediate neighbourhood of an individual).

Finally, the available Bikeability delivery data, including the month in which training took place, were merged into the MCS data using the Unique Reference Number of each child׳s current school. This allowed us to identify children whose school had offered Bikeability training before the date of the MCS survey interview. Children were classed as ‘uncertain’ with regard to their cycle training status if it was not possible to tell whether or not they had been offered Bikeability in school at the time of the MCS survey. This uncertainty could arise if their school had offered Bikeability training in the same month as their MCS interview but, because the day of delivery was not recorded in the operational data, it was unclear which had happened first (*N*=192). It could also arise if their school had offered training both before and after the interview (*N*=195) or if the operational Bikeability data was missing data on the month in which training took place (*N*=56).

#### 2.2.4 Statistical analyses

After first conducting descriptive analyses, we fit Poisson regression models with robust standard errors, in which the outcome was whether the parent reported that the child had completed cycle training. We initially fit minimally-adjusted models, adjusting only for the child׳s region of England and the month of data collection. We then fit models that adjusted for all child, family and area characteristics (adjusted model 1). Finally, we additionally adjusted for whether the child׳s school had offered Bikeability (adjusted model 2). We fit this final adjusted model in order to examine how far any observed individual-level differences between groups of children might be explicable in terms of school-level differences with regard to offering Bikeability.

We initially fit models using the total study population, and then stratified the models according to whether the child׳s school had offered Bikeability prior to the date of the MCS interview. We performed this stratification in order to examine whether the predictors of participating in cycle training varied according to whether the school had offered Bikeability, and we also used tests for interaction to test for this formally. In this way, we sought to generate evidence concerning the extent to which any observed inequalities in uptake rate between groups of children might be mitigated by offering Bikeability in their school. All stratified models were adjusted for the characteristics presented in Table 2, plus the region in England where the child lived and the month of MCS data collection.

The proportion of missing data was 3% for weight status, 9% for whether the child cycled to school at age 7, and <0.2% for all other variables presented in Table 2. Missing data were imputed using multiple imputation by chained equations (five imputations) under an assumption of missing at random. Using the ‘svyset’ commands in Stata, all regression analyses allowed for the stratified sampling design used in the first sweep of MCS; allowed for clustering of children within schools (*N*=3116 unique schools for the 6986 participants); and used the fifth-sweep MCS weights assigned to each child to allow for differences in sampling rates, response rates and follow-up rates across strata. Our substantive findings were very similar when we instead fit multi-level logistic regression models of children nested within schools.

## 3 Results

### 3.1 Which schools offer Bikeability?

**Fig. 1:**
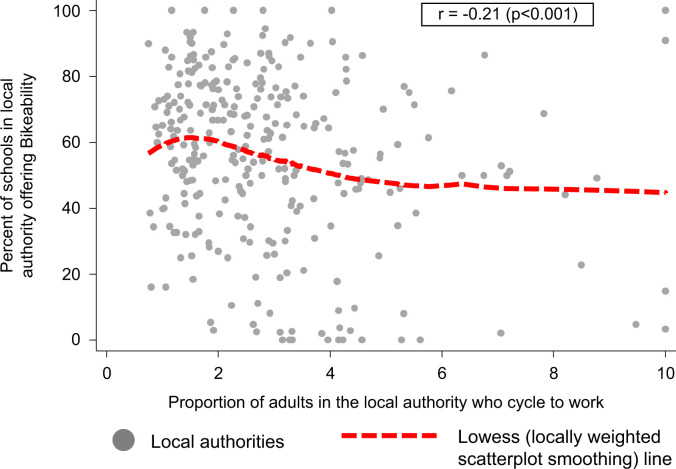
Scatter graph showing the association between the prevalence of adult cycling and the proportion of schools offering Bikeability, across 293 non-London, English local authorities. Locally weighted scatterplot smoothing (‘lowess’) line fitted using running-line least-squares smoothing, based on 293 local-authorities.

An estimated 55% of English primary schools outside London (containing 57% of children) offered Bikeability cycle training to the cohort of children who left primary school in 2012. At the regional level these proportions showed relatively modest variation (ranging from 44% in the North East to 61% in the North West and West Midlands), but at the level of local authorities the proportions spanned the whole range from 0% to 100% (see Fig. 1 and Supplementary Fig. S1).

As shown in Table 1, the smallest 20% of schools were somewhat less likely to offer Bikeability than their larger counterparts, as were schools outside large urban areas. Schools were also less likely to offer Bikeability as the level of deprivation among the student body increased. Finally, there was a marginally-significant trend for schools to offer Bikeability less often as the local prevalence of adult cycling increased. A similar trend was seen at the local authority level; local authorities with a higher overall prevalence of cycling tended to contain a somewhat smaller proportion of schools offering Bikeability (Table 1, see also Fig. 1).

### 3.2 Which children participate in cycle training?

Overall, parents of 47% of our MCS study population reported that their child had participated in formal cycle training. This proportion was substantially higher among children whose schools had offered Bikeability training during the relevant time period than among children whose school had not (68% vs. 34%). Table 2 examines which other characteristics were associated with participating in cycle training among the full MCS study population. Table 3 presents equivalent analyses stratified by whether the child׳s school had offered Bikeability prior to the MCS interview, in order to examine how far offering Bikeability in schools might alter the nature of the associations seen.

#### 3.2.1 Cycle training participation by age, sex and ethnicity

There were no overall differences in cycle training participation rates by sex or age (Table 2). There was, however, evidence of an interaction between sex and whether the school offered Bikeability. Specifically, girls were less slightly likely than boys to have participated in cycle training in schools that had not offered Bikeability, whereas a trend towards the reverse association was apparent in schools that had offered Bikeability (Table 3).

There was strong evidence of a difference in cycle training participation across ethnic groups, with markedly lower rates of participation among South Asian and ‘Other’ ethnicity children than among White children, and slightly lower rates among mixed ethnicity and Black children. This association was somewhat attenuated but remained strong after adjusting for other child, family and area characteristics (Adjusted model 1). It likewise remained strong after additionally adjusting for whether the school had offered Bikeability training (Adjusted model 2), suggesting that these ethnic differences could not be explained solely in terms of minority ethnic children attending schools that were less likely to offer Bikeability. Further confirmation of this point is provided in Table 3, which shows that a trend towards lower rates of uptake was seen in minority ethnic children regardless of whether their school had offered Bikeability. Yet while an association in the same direction was seen in both strata, tests for interaction provided strong evidence that the magnitude of the association differed between these two types of school. Specifically, there was strong evidence that these ethnic differences were more pronounced among children whose schools had not offered the training (e.g. the adjusted risk ratio for South Asian vs. White children was 0.53 (95% CI 0.39, 0.71) in schools that did not offer Bikeability training vs. only 0.80 (95% CI 0.65, 0.97) in schools that did offer Bikeability).

#### 3.2.2 Cycle training participation by health and physical activity characteristics

In minimally-adjusted analyses there was evidence that children who were overweight/obese or who had poorer health were less likely to have participated in cycle training. These differences were, however, attenuated to the null after adjustment for parental education and income (all *p*>0.05 in Adjusted model 1, and also in models adjusting only for these two parental characteristics [data not shown]).

There was strong evidence of higher rates of cycle training participation among children who took part more frequently in clubs or classes for sport or other types of physical activity, and this was only partially attenuated after adjusting for other child and family characteristics. This association was observed regardless of whether the child had been offered Bikeability in school, but the association was stronger among children who had not been offered Bikeability (*p*=0.01 for interaction, see Table 3). Lastly, there was a trend towards higher rates of cycle training participation among children who had cycled to school at age 7 but this was not significant; the very small number of children who cycled to school at age 7 meant that these analyses were considerably underpowered.

#### 3.2.3 Cycle training participation by socio-economic and area characteristics

Rates of participation in cycle training were lower among children of less educated or less affluent parents, with this association being particularly marked among the least educated and poorest groups. The association with parental income was attenuated slightly after adjusting for whether the school had offered Bikeability (Adjusted model 2 in Table 2), suggesting that some of the observed association reflected a tendency of children in the least educated and poorest groups to attend schools that did not offer Bikeability. Nevertheless, even after adjusting for whether the school had offered Bikeability, significant associations with parent socio-economic position remained. Particularly for parental education, there was again some evidence that the magnitude of these differences was greater among children in schools which had not offered Bikeability (*p*=0.02 for interaction).

It is worth noting that the effect sizes associated with low parental education and income were somewhat larger in the sample of children whose schools had not offered Bikeability than in the total sample (final column of Table 3 vs. Adjusted model 1 in Table 2). This suggests that overall the Bikeability programme narrowed somewhat these socio-economic inequalities in participation rates in cycle training. In other words, although the Bikeability programme may have tended to increase between-school inequalities because it was offered less often in the poorest schools, this effect seems to have been more than offset by its role in reducing within-school socio-economic inequalities in participation rates.

Finally, there was no evidence that rates of participation in cycle training differed systematically according to the prevalence of cycle commuting in the local area. There was evidence in minimally adjusted analyses of lower participation rates in large urban areas, but this association was attenuated to the null after adjustment for child and family characteristics.

## 4 Discussion

In our sample of 6986 English children who left primary school in 2012, cycle training participation rates were lower among minority ethnic children, among children who played sport less often, and among children whose parents were poorer or less educated. All these trends were observed regardless of whether the school offered Bikeability cycle training, but the associations were stronger among children whose school had not offered Bikeability. These findings therefore suggest that offering Bikeability in schools reduces, but does not eliminate, overall inequalities in participation rates in cycle training among children. Moreover, our school-level analyses also indicate that the reduction in socio-economic inequalities was partly offset by the fact that the Bikeability programme was itself offered less often by the schools with the most deprived student body.

### 4.1 Strengths and limitations

A key strength of this study lies in its use of an existing British cohort study as a platform for examining a specific national policy measure. This allowed a more detailed examination of participation in cycle training than would have been possible using other data sources (e.g. using school-level delivery data alone), while also being much less expensive than conducting a new, primary study on the same scale. We were able to use the existing structure of MCS in this way by collaborating with the Department for Transport to commission one bespoke survey question (on cycle training) and then merging in operational Bikeability data. We hope that more studies in the future may use similar approaches to maximise the research potential of established cohorts or routine national surveys (Craig et al., 2012, Goodman et al., 2013).

Nevertheless our study also has some important limitations, many of which are related to our reliance on data from a national cohort with very broad research aims rather than a specific focus on cycling. Parents were asked only a single cycle training question, which prevented us from distinguishing Bikeability from non-Bikeability cycle training, or cycle training delivered in school from that delivered out of school. We were likewise only able to ask parents about the child׳s participation in cycle training and could not ask the children or their teachers. This is likely to have introduced some measurement error, although it is somewhat reassuring that the proportion of parents in our study reporting cycle training was 47%, which is similar to the estimate that half of children nationally take part in Bikeability.

Similarly, although our triangulation of MCS data with Bikeability delivery data is a strength, our reliance upon operational Bikeability data limited us somewhat in terms of coverage (notably, with London being excluded) and in terms of temporal resolution (with delivery data available at most to the nearest month). The fact that the MCS data and Bikeability delivery data came from different sources also meant that we were not able to classify children according to whether they themselves had been offered Bikeability training, but only as to whether their school had offered it to their year group. This likewise introduces some measurement error, although we believe this is unlikely to have a large effect on our substantive findings since most schools offering Bikeability do offer it to an entire year group.

Finally, the scope of our study is limited in its focus on a specific programme in a single country. We believe that Bikeability is worthy in its own right of such in-depth investigation because it is one of the best-funded and most widely-implemented government initiatives aimed at encouraging cycling in England. Bikeability is also potentially of wider international interest as a relatively rare example of a child cycle training scheme that is funded and coordinated nationally, rather than locally or regionally, and that schools across the whole country are encouraged to deliver. In recent years several other countries have expressed interest in replicating this national delivery model (Department for Transport, personal communication). As we outline below, some of our broader findings may hold lessons both for these countries and for other cycling programmes. Nevertheless, it is unclear how many of the details of our findings will generalise to other initiatives or other settings, and seeking to examine this would therefore be a useful direction for future work.

### 4.2 Interpretation of the study and implications for future research

Our finding of socio-economic and ethnic differences in cycle training participation rates resonates with other fields of public health and health service research (Marmot, 2010) in illustrating that offering initiatives free at the point of delivery does not guarantee equitable uptake. This study also highlights how such inequalities can occur at multiple levels, with school-level socio-economic variation in Bikeability delivery rates compounding child-level socio-economic variation in participation rates. Reducing the resulting socio-economic inequalities in participation in cycle training is arguably a particular priority given that children from poorer backgrounds are at the highest risk of cycling injury (Edwards et al., 2006). Some reduction in these inequalities may already have occurred following the introduction in 2012/13 of Bikeability-funded ‘pool bikes’ that schools can lend to children who lack a bike in sufficiently good condition (Department for Transport, 2012b). Evaluating how far this initiative has in fact increased uptake in schools serving more deprived students will be one useful line of further research; to the extent that these pool bikes are successful in widening access to the programme, it may be a useful model for other countries to follow. More broadly, our finding of inequalities at multiple levels points to the potential importance of cycling initiatives seeking to tackle barriers to cycling ‘upstream’, at the level of areas and institutions (Lorenc et al., 2013). Such initiatives may be most effective at reducing inequalities when they include some targeting of more deprived areas or schools, as has been attempted with apparent success in the English ‘Cycling Towns’ programme (Goodman et al., 2013) and the American ‘Safe Routes to School’ programme (McDonald et al., 2013).

As for the finding that minority ethnic, and particularly South Asian, children are less likely to have completed cycle training, this is interesting in that it mirrors ethnic variation in cycling levels among adults (e.g. 1.4% of South Asian commuters cycled to work in 2011, compared to 3.1% of White British adults (Office for National Statistics, 2014)). Qualitative research suggests that some minority ethnic groups, including South Asians, may consider cycling a mode of transport that is unappealing or even irrelevant for ‘people like them’ (Steinbach et al., 2011). It is plausible that this reduces parental motivation to ensure that their children have good cycling skills. For policymakers wishing to increase the ethnic diversity of cyclists in the future, however, formal cycle training may be particularly important for those ethnic groups where parents are less likely to be able to teach skills themselves. The same may plausibly apply in high-cycling countries like the Netherlands, where levels of cycling are fairly equitable by gender and across socio-economic groups but are considerably lower among minority ethnic individuals (Martens, 2013, Mäki-Opas et al., 2014).

Encouragingly, offering Bikeability in school was associated with increased participation in cycle training across all types of children and with reduced socio-economic and ethnic differences. This suggests that these differences do not necessarily reflect active parental objections to cycle training, and that encouraging more schools to offer Bikeability will go some way towards reducing overall inequalities. We think it plausible that this finding that in-school delivery increased participation and reduced inequalities may generalise to other high-income countries with comparable cycling levels. To the extent that this is the case, it strengthens the argument for a centrally-funded and coordinated national cycle training scheme that is promoted to all schools. This contrasts with the current situation in most countries, in which access to schemes varies across regions, or there is more onus on schools and children to seek out training. Our findings also indicate, however, that simply offering Bikeability in school does not eliminate these inequalities. This highlights the potential value of further research into specific socio-economic and ethnic barriers to Bikeability cycle training. It also suggests the potential value of considering whether such barriers may operate with respect to other cycling initiatives that are also free at the point of delivery in the UK, such as adult cycle training. By contrast, offering Bikeability in school did seem to be sufficient to eliminate (and, in fact, weakly reverse) the tendency that was otherwise observed for boys to complete cycle training more often than girls.

The third strong predictor of cycle training participation was how often the child participated in sport, providing an intriguing hint that children may themselves be exercising preferences as to whether they receive training. This may reflect the fact that, whereas most schools provide the training as a default activity for all children, some schools offer it to children on a more explicitly ‘opt-in’ basis. By contrast, it was notable that weight status, poor health and long-term illness were generally not associated with participating in cycle training. Taken together, this suggests that cycle training appears currently to be fairly inclusive with regard to physical health status and disability, but it might be useful to consider how to make it more appealing to less physically active children. Further consideration of this last point is also potentially important, insofar as the least active children are also plausibly those with most to gain from increasing their cycling levels. We hypothesise that one barrier for less sporty children may be a lack of familiarity or confidence in the physical act of riding a bicycle. In this respect, the UK plausibly contrasts with the Netherlands in which most children can ride a bicycle before they start school. If this is true, one useful intervention would be to teach children these prerequisite motor skills from an early age – for example, by teaching children how to ride a bicycle (in a fully off-road environment) in physical education lessons in the early years of primary school.

Finally, it is perhaps surprising that participation in cycle training does not vary according to the local prevalence of cycling and, indeed, that school uptake of Bikeability appears if anything to be lower in areas with a high cycling prevalence. The reasons for this are not clear, since local authorities can access central government funding for Bikeability and therefore rarely run their own cycle training programmes instead. Speculatively, it is possible that some high-cycling local authorities may feel that cycle training is not needed because children already receive sufficient instruction from their parents or from other sources such as holiday camps. If this is the case, then the evidence presented here regarding socio-economic and ethnic inequalities in cycle training participation rates may help persuade them of the value of offering this training to all schoolchildren.

In conclusion, successfully promoting the Bikeability scheme to a larger number of local authorities, schools and parents would be expected not only to increase the proportion of children receiving formal cycle training but also to reduce (although not necessarily eliminate) current inequalities in participation rates. Such promotion of the scheme should be coupled with further research into specific barriers which some types of schools, parents or children may face with respect to cycle training. It should also be coupled with further evaluation of the ultimate impacts of the scheme upon cycling skills and behaviour, and we hope in future research to use the Millennium Cohort Study to contribute to the evidence base in this latter respect. By triangulating research in these ways, it is to be hoped that Bikeability can maximise its ability to realise its goal to get more children cycling more safely and more often.

## Funding

This study was funded by the Economic and Social Research Council (ESRC: Grant no. ES/L013606/1). DO and EvS are also supported by the Medical Research Council (Unit Programme numbers MC_UU_12015/6; MC_UU_12015/7). The views presented here are those of the authors, and do not necessarily reflect those of the ESRC or the Department for Transport. Colleagues at the Department for Transport collaborated to commission the cycling-related questions in MCS. Otherwise neither the ESRC nor the Department for Transport played any role in the study design, analysis and interpretation of data, writing of the report or the decision to submit the article for publication.

## Figures and Tables

**Table 1:** School-level analysis: predictors of offering Bikeability training among English schools outside of London (*N*=12,881 schools).

Variable	Level	*N* (%) of schools	% Offering Bikeability	Minimally-adjusted risk ratio (95% CI)	Adjusted risk ratio (95% CI)
School size: number of pupils in Years 5 and 6	Fifth 1 (smallest)	2434	47	1⁎⁎⁎	1⁎⁎⁎
Fifth 2	2591	55	1.16 (1.08, 1.24)	1.15 (1.08, 1.23)
Fifth 3	2349	59	1.23 (1.13, 1.33)	1.17 (1.09, 1.27)
Fifth 4	2829	58	1.23 (1.13, 1.33)	1.17 (1.08, 1.26)
Fifth 5 (largest)	2608	58	1.20 (1.10, 1.32)	1.12 (1.03, 1.23)
Per cent students receiving free school meals	<10%	5604	58	1⁎	1⁎⁎⁎
10–19.9%	3057	57	0.99 (0.95, 1.03)	0.95 (0.91, 0.99)
20–29.9%	1718	53	0.91 (0.84, 0.99)	0.86 (0.79, 0.92)
30–39.9%	1209	52	0.88 (0.80, 0.97)	0.82 (0.74, 0.90)
≥40%	1223	48	0.80 (0.71, 0.91)	0.74 (0.66, 0.84)
Settlement type	Large urban	8374	58	1⁎⁎	1⁎⁎⁎
Small town and fringe	1485	53	0.94 (0.86, 1.02)	0.86 (0.79, 0.93)
Village or smaller	2952	50	0.87 (0.80, 0.95)	0.84 (0.77, 0.92)
Prevalence of adult cycling in the local area	<2%	6619	57	1	1⁎
2–3.9%	4133	55	0.97 (0.90, 1.05)	0.95 (0.88, 1.03)
4–5.9%	1165	51	0.89 (0.80, 0.99)	0.86 (0.77, 0.96)
≥6%	894	47	0.84 (0.68, 1.02)	0.80 (0.65, 0.98)

Minimally-adjusted analyses adjusted for the school׳s region in England, adjusted analyses additionally adjusted for all variables in column.

⁎ *p*<0.05, in tests for heterogeneity.

⁎⁎ *p*<0.01, in tests for heterogeneity.

⁎⁎⁎ *p*<0.001, in tests for heterogeneity.

**Table 2:** Child-level analyses: predictors of completing cycle training among the full sample of Millennium Cohort Study children (*N*=6986).

Variable	Level	*N*	% Done cycle training	Risk ratio for cycle training (95% CI)		
Minimally-adjusted	Adjusted 1	Adjusted 2
Sex	Female	3471	47	1	1	1
Male	3515	48	1.01 (0.96, 1.07)	1.01 (0.95, 1.07)	1.01 (0.96, 1.07)
Age	10 years	2574	45	1	1	1
11 years	4412	48	1.02 (0.96, 1.08)	1.02 (0.96, 1.08)	1.00 (0.94, 1.06)
Ethnicity	White	5726	52	1⁎⁎⁎	1⁎⁎⁎	1⁎⁎⁎
Mixed	225	43	0.88 (0.74, 1.05)	0.92 (0.78, 1.10)	0.93 (0.79, 1.10)
South Asian	860	23	0.51 (0.42, 0.61)	0.63 (0.51, 0.76)	0.67 (0.56, 0.80)
Black	104	41	0.82 (0.56, 1.20)	0.89 (0.61, 1.30)	0.94 (0.64, 1.39)
Other	69	23	0.48 (0.27, 0.84)	0.56 (0.33, 0.94)	0.57 (0.35, 0.93)
Weight status	Normal/underweight	4968	48	1⁎	1	1
Overweight	1383	46	0.92 (0.86, 0.99)	0.95 (0.89, 1.01)	0.96 (0.90, 1.02)
Obese	416	43	0.91 (0.81, 1.03)	1.00 (0.89, 1.14)	0.99 (0.87, 1.11)
General health	Good/excellent	6735	48	1⁎⁎⁎	1	1
Fair/poor	246	31	0.72 (0.59, 0.87)	0.86 (0.70, 1.04)	0.90 (0.75, 1.09)
Longstanding illness	No	5965	48	1⁎	1	1
Yes	1013	44	0.89 (0.82, 0.97)	0.93 (0.86, 1.01)	0.93 (0.86, 1.01)
Frequency of attending club or classes for sport or exercise	Not at all	1810	35	1⁎⁎⁎	1⁎⁎⁎	1⁎⁎⁎
≤1 time a week	1661	46	1.30 (1.19, 1.42)	1.23 (1.13, 1.35)	1.24 (1.14, 1.35)
2–3 times a week	2383	53	1.45 (1.34, 1.58)	1.29 (1.19, 1.41)	1.30 (1.20, 1.41)
4–5 times a week	1130	56	1.49 (1.36, 1.64)	1.29 (1.17, 1.41)	1.33 (1.22, 1.46)
Cycled to/from school age 7	No	6295	48	1	1	1
Yes	68	57	1.21 (0.94, 1.57)	1.11 (0.86, 1.44)	1.07 (0.86, 1.35)
Highest education of either parent†	Degree	1032	53	1⁎⁎⁎	1⁎⁎	1⁎⁎
Diploma	2444	53	1.00 (0.92, 1.07)	1.02 (0.94, 1.10)	1.02 (0.95, 1.10)
Higher secondary	1040	49	0.91 (0.83, 1.01)	1.00 (0.91, 1.11)	1.00 (0.90, 1.10)
Middle secondary	1475	44	0.81 (0.73, 0.89)	0.92 (0.83, 1.02)	0.92 (0.83, 1.02)
Low/other/none	984	31	0.61 (0.54, 0.70)	0.80 (0.70, 0.93)	0.82 (0.71, 0.94)
Equivalised household income‡	Fifth 1 (highest)	1293	55	1⁎⁎⁎	1⁎⁎	1⁎
Fifth 2	1414	56	0.95 (0.88, 1.03)	0.98 (0.91, 1.06)	0.98 (0.91, 1.05)
Fifth 3	1407	50	0.84 (0.78, 0.91)	0.91 (0.83, 0.99)	0.91 (0.84, 0.99)
Fifth 4	1395	45	0.77 (0.70, 0.85)	0.91 (0.82, 1.01)	0.92 (0.84, 1.02)
Fifth 5 (lowest)	1477	32	0.58 (0.52, 0.66)	0.78 (0.68, 0.89)	0.83 (0.73, 0.94)
Prevalence of cycling to work in local area	< 2%	3493	45	1	1	1
2–3.9%	2285	49	1.06 (0.98, 1.15)	1.07 (0.99, 1.15)	1.04 (0.97, 1.12)
4–5.9%	706	51	1.13 (1.01, 1.27)	1.15 (1.02, 1.30)	1.13 (1.02, 1.26)
6–9.9%	428	48	1.08 (0.91, 1.27)	1.09 (0.93, 1.28)	1.09 (0.95, 1.26)
≥10%	69	49	0.97 (0.71, 1.34)	0.95 (0.70, 1.30)	0.97 (0.73, 1.28)
Settlement type	Large urban	5619	46	1⁎⁎	1	1
Small town and fringe	654	51	1.11 (0.97, 1.27)	1.04 (0.91, 1.19)	1.09 (0.96, 1.25)
Village or smaller	691	55	1.16 (1.05, 1.29)	1.07 (0.96, 1.18)	1.10 (1.00, 1.21)
School offered Bikeability prior to interview	Yes	2563	68	1⁎⁎⁎		1⁎⁎⁎
No	3980	34	0.51 (0.47, 0.56)		0.53 (0.49, 0.57)
Uncertain	443	49	0.74 (0.66, 0.84)		0.75 (0.67, 0.85)

CI=confidence intervals.

⁎⁎⁎ *p*<0.001, with *p*-values for heterogeneity.

⁎⁎ *p*<0.01, with *p*-values for heterogeneity.

⁎ *p*<0.05, with *p*-values for heterogeneity.

† Includes both academic and vocational qualifications. ‘Degree’ corresponds to British National Vocational Qualification (NVQ) level 1, ‘Diploma’ to NVQ2, ‘Higher secondary’ to NVQ3, ‘Middle Secondary’ to NVQ2 and ‘Low, other or none’ to NVQ1, overseas qualifications or no qualifications.

‡ Equivalised for household composition in terms of adults and children (Hansen, 2014). Minimally-adjusted analyses adjusted for the child׳s region of England and the month of data collection, adjusted models additionally adjusted for all variables in the column.

**Table 3:** Child-level analyses: predictors of completing cycle training, stratified by whether the school had offered Bikeability (*N*=6543)

Variable	Level	Bikeability offered in school			Bikeability not offered in school			Interaction with school offering Bikeability
*N*	% Done cycle training	Adjusted RR for cycle training (95% CI)	*N*	% Done cycle training	Adjusted RR for cycle training (95% CI)
Sex	Female	1301	68	1	1975	32	1⁎	0.01
Male	1262	67	0.95 (0.89, 1.01)	2005	35	1.11 (1.00, 1.22)	
Age	10 years	848	69	1	1545	32	1	0.21
11 years	1715	67	1.00 (0.94, 1.07)	2435	35	1.01 (0.91, 1.13)	
Ethnicity	White	2213	70	1	3124	39	1⁎⁎⁎	0.008
Mixed	78	72	1.04 (0.87, 1.24)	136	26	0.79 (0.57, 1.12)	
South Asian	219	47	0.80 (0.65, 0.97)	605	13	0.53 (0.39, 0.71)	
Black	33	67	0.87 (0.62, 1.22)	68	29	1.02 (0.51, 2.05)	
Other	20	55	0.77 (0.43, 1.37)	45	9	0.37 (0.13, 1.09)	
Weight status	Normal/underweight	1824	69	1	2830	35	1	0.70
Overweight	496	69	0.98 (0.91, 1.05)	799	32	0.91 (0.81, 1.03)	
Obese	154	60	0.91 (0.78, 1.05)	233	31	1.03 (0.84, 1.27)	
General health	Good/excellent	2492	68	1	3809	34	1	0.37
Fair/poor	71	51	0.99 (0.77, 1.28)	166	22	0.82 (0.61, 1.11)	
Longstanding illness	No	2202	69	1⁎	3380	34	1	0.11
Yes	359	60	0.89 (0.81, 0.99)	594	35	1.02 (0.90, 1.16)	
Frequency of attending club or classes for sport or exercise	Not at all	655	54	1⁎⁎⁎	1043	23	1⁎⁎⁎	0.01
≤1 time a week	602	69	1.20 (1.09, 1.33)	951	33	1.33 (1.14, 1.55)	
2–3 times a week	901	74	1.24 (1.13, 1.36)	1325	37	1.41 (1.21, 1.64)	
4–5 times a week	405	75	1.26 (1.14, 1.39)	659	46	1.49 (1.27, 1.76)	
Cycled to/from school age 7	No	2324	69	1⁎⁎	3572	35	1	0.23
Yes	26	92	1.29 (1.07, 1.55)	30	30	0.88 (0.56, 1.40)	
Highest education of either parent	Degree	410	70	1⁎	556	40	1⁎	0.02
Diploma	914	74	1.06 (0.98, 1.16)	1374	39	0.99 (0.87, 1.13)	
Higher secondary	385	73	1.11 (0.99, 1.24)	581	33	0.91 (0.76, 1.08)	
Middle secondary	545	61	0.94 (0.85, 1.06)	836	33	0.93 (0.78, 1.11)	
Low/other/none	304	54	0.96 (0.83, 1.12)	627	19	0.69 (0.53, 0.88)	
Equivalised household income	Fifth 1 (highest)	500	73	1⁎	708	42	1	0.09
Fifth 2	567	75	0.99 (0.91, 1.08)	747	42	0.98 (0.86, 1.12)	
Fifth 3	565	70	0.94 (0.86, 1.03)	749	35	0.84 (0.72, 0.99)	
Fifth 4	510	64	0.91 (0.81, 1.02)	802	33	0.92 (0.76, 1.12)	
Fifth 5 (lowest)	421	53	0.82 (0.71, 0.95)	974	22	0.79 (0.63, 1.00)	
Prevalence of cycling to work in local area	< 2%	1202	68	1	2084	32	1	0.73
2–3.9%	892	68	1.03 (0.96, 1.10)	1220	35	1.05 (0.92, 1.19)	
4–5.9%	271	68	1.05 (0.95, 1.16)	398	40	1.26 (1.03, 1.53)	
6–9.9%	173	65	1.03 (0.87, 1.22)	238	35	1.14 (0.89, 1.47)	
≥10%	25	72	0.96 (0.69, 1.34)	39	31	0.87 (0.51, 1.49)	
Settlement type	Large urban	2121	66	1	3135	32	1	0.77
Small town and fringe	200	70	1.01 (0.89, 1.14)	417	41	1.09 (0.87, 1.37)	
Village or smaller	237	79	1.09 (1.00, 1.19)	415	41	1.06 (0.89, 1.27)	

RR=risk ratio, CI=confidence interval. 443 children excluded from these analyses because of uncertainty as to whether they had been offered Bikeability at the time of interview. Adjusted models adjusted for all variables in the column, plus the child׳s region of England and the month of data collection. Interaction *p*-value presented from adjusted models; *p*-values from unadjusted models were very similar.

⁎⁎⁎ *p*<0.001, with *p*-values for heterogeneity.

⁎⁎ *p*<0.01, with *p*-values for heterogeneity.

⁎ *p*<0.05, with *p*-values for heterogeneity.
