# Supplementary material for: Cycle training for children: Which schools offer it and who takes part?
Source: J Transp Health. 2015 Dec;2(4):512–21. doi: 10.1016/j.jth.2015.07.002 (PMC4728135; doi:10.1016/j.jth.2015.07.002)

**Supplementary material to “Cycle training for children: which schools offer it and who takes part?”**

**Supplementary methods: identifying schools that offered Bikeability**

We sought to identify all schools that had offered Bikeability cycle training to the cohort of children leaving primary school in 2012, using operational data provided by the Department for Transport. Schools are encouraged to deliver Bikeability in the final year of primary school (Year 6, age 10-11), but a minority instead deliver the training a year earlier (Year 5, age 9-10). We therefore sought to identify all schools that had offered cycle training to the 2012 cohort of interest at some point during their final two years of school. We did this by identifying schools that either offered Bikeability to Year 5 children in the academic year 2010/11 or offered Bikeability to Year 6 children in the academic year 2011/12.

Unfortunately, the Department for Transport collects some delivery data using financial years (which run from April to March) rather than academic years (which run from September to August), and the Department did not routinely collect data on the month of delivery until April 2011. This meant that for a proportion of Year 5 delivery, we could identify schools that had delivered Bikeability between April 2010 and March 2011, but could not tell if the delivery had occurred between April 2010 and August 2010 (i.e. too soon to affect our MCS cohort) or between September 2010 and March 2011 (i.e. in the right academic year to affect our MCS cohort).

Throughout the analyses presented in this paper we assumed that these ambiguous schools did in fact offer Bikeability to our cohort members – i.e. our definition of which schools offered Bikeability was somewhat over-inclusive. Few schools were, however, affected by this decision. For example, removing these ambiguous schools reduced our estimate of the proportion of schools offering Bikeability from 55% to 51%. All our findings were very similar in a sensitivity analysis that assumed that these ambiguous schools did not offer Bikeability to our cohort members.

**Supplementary results: proportion of schools offering Bikeability across local authorities**

Supplementary Figure S1: Map showing the proportion of schools offering Bikeability training across 293 non-London, English local authorities.


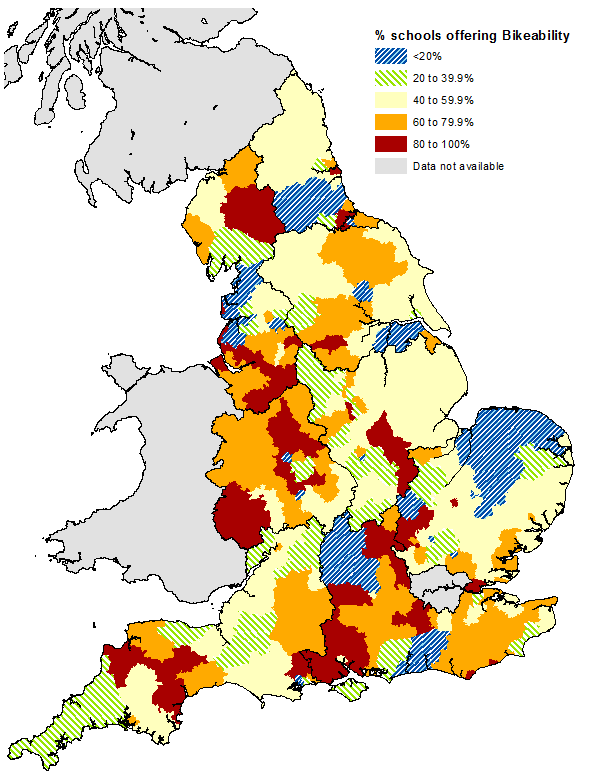

Supplement: Supplementary file 1 — Supplementary data [file mmc1.doc]
